# Seropositive Rheumatoid Arthritis Emerging During Acute Phase of Lung Abscess: A Case Report

**DOI:** 10.1002/rcr2.70602

**Published:** 2026-04-29

**Authors:** Yusuke Arai, Hiromu Tanaka, Kazuoto Hiramoto, Aina Nomura, Takahiro Asami, Takashi Inoue

**Affiliations:** ^1^ Department of Internal Medicine Sano Kosei General Hospital Tochigi Japan; ^2^ Division of Rheumatology, Department of Medicine Keio University School of Medicine Tokyo Japan

**Keywords:** acute phase, bacterial pneumonia, polyarthralgia, respiratory infections, seropositive rheumatoid arthritis

## Abstract

Rheumatoid arthritis (RA) is an autoimmune disease where immunological activation often precedes clinical onset. Although respiratory infections are recognized risk factors for RA, the onset of RA shortly after acute infection remains poorly characterized. Herein, we report a rare case of seropositive RA that became clinically apparent during the acute phase of lung infection. An 87‐year‐old man who had never smoked was diagnosed with a lung abscess caused by methicillin‐susceptible *Staphylococcus aureus* . Post discharge, he developed polyarthritis, and laboratory tests revealed positive results for rheumatoid factor (RF) and anti‐citrullinated protein antibodies (ACPA). He was diagnosed with seropositive RA, and his symptoms improved with corticosteroid therapy. Retrospective review of positron emission tomography scans performed before admission revealed inflammatory uptake in multiple joints, suggesting a preclinical phase. This case suggests that acute respiratory infection may accelerate the transition from latent autoimmunity to clinically overt RA, highlighting the diagnostic value of RF and ACPA testing in new‐onset arthritis after infection.

## Graphical abstract

We report a rare case of seropositive rheumatoid arthritis (RA) that became clinically apparent during the acute phase of lung infection. This case demonstrates that RA can be diagnosed during the acute course of a respiratory infection and highlights the diagnostic value of rheumatoid factor and anti‐citrullinated protein antibodies testing when new arthritis develops.

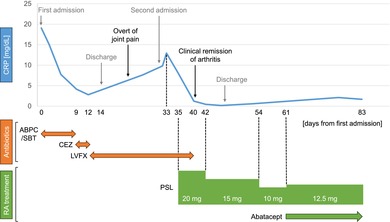

## 1 Introduction

Rheumatoid arthritis (RA) is a chronic autoimmune disease characterized by synovitis and progressive joint destruction. Increasing evidence supports the ‘mucosal origins hypothesis’, proposing that RA may arise from immune dysregulation at mucosal sites, with the lung considered a key organ [1]. Seropositive preclinical phases characterized by anti‐cyclic citrullinated peptide antibody (ACPA) and rheumatoid factor (RF) further indicate that immunological activation precedes clinical onset.

Respiratory infections and chronic airway inflammation are associated with an increased risk for RA. However, a previous study typically reported a prolonged interval of 5 years between respiratory infection and RA development [2], and RA diagnosed shortly after acute respiratory infection remains poorly characterized. Moreover, post‐infectious arthritis is often difficult to distinguish from other joint disorders, potentially delaying the diagnosis.

Herein, we report a rare case in which seropositive RA was clinically diagnosed during the acute phase of a lung abscess, suggesting that respiratory infection may act as an immunological trigger, and highlight the diagnostic value of RF and ACPA testing in patients with new‐onset arthritis after infection.

## 2 Case Report

**FIGURE 1:**
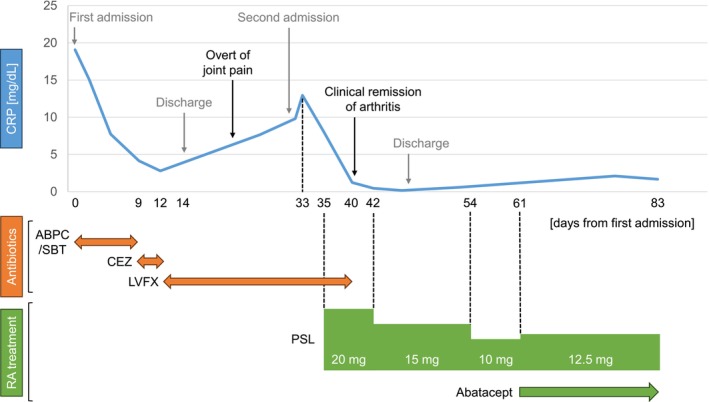
Clinical course and treatment timeline. Changes in serum C‐reactive protein (CRP) levels over time are associated with clinical events and treatment. ABPC/SBT, ampicillin/sulbactam; CEZ, cefazolin; LVFX, levofloxacin; PSL, prednisolone.

An 87‐year‐old man who had never smoked presented with a 1‐week history of anorexia, fatigue and fever that began the day before referral. Chest computed tomography (CT) revealed consolidation in the right lower lobe, and sputum cultures exhibited methicillin‐susceptible *Staphylococcus aureus* . Although the patient had no respiratory failure, laboratory tests revealed marked inflammation with a C‐reactive protein level of 19.07 mg/dL. The patient was diagnosed with bacterial pneumonia that improved with antibiotic therapy. Follow‐up CT revealed partial lung abscess formation, for which prolonged antibiotic therapy was prescribed, and the patient was discharged with oral antibiotics (Figure 1).

**FIGURE 2:**
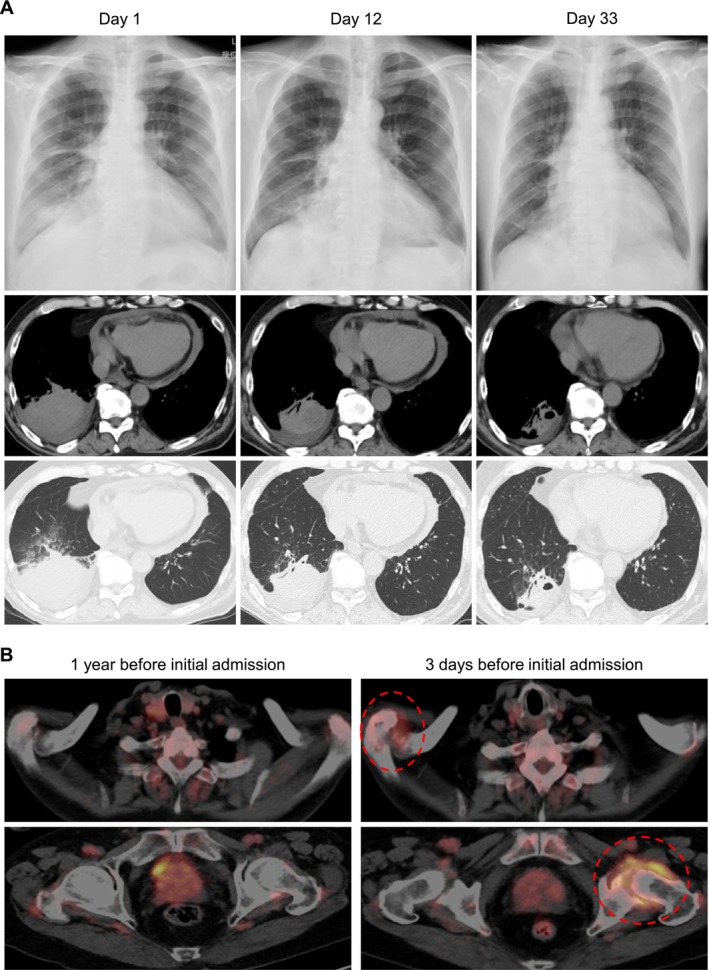
Serial radiological findings and FDG‐PET/CT images. (A) Chest radiographs obtained on Day 1, Day 12 and Day 33 demonstrate gradual improvement of right lower lung opacity. Axial chest CT images show consolidation with pleural effusion in the right lower lobe on Day 1, consistent with lung abscess formation, followed by progressive reduction on Day 12 and Day 33. Corresponding lung window images reveal residual organizing changes during the recovery phase. (B) FDG‐PET/CT images obtained 1 year before admission and 3 days before initial admission demonstrate newly developed focal fluorodeoxyglucose uptake in the right shoulder and left hip joints (red dashed circles), findings that were absent on the previous study and are suggestive of preclinical inflammatory arthritis. CT, computed tomography; FDG, fluorodeoxyglucose; PET, positron emission tomography.

He reported history of mild chronic right shoulder pain without swelling for several months. Post discharge, his shoulder pain worsened and a new painful swelling developed in the right wrist and both knees. He was initially treated with nonsteroidal anti‐inflammatory drugs for suspected pseudogout in an outpatient setting. However, his symptoms progressed, resulting in impaired mobility and readmission. Repeated laboratory tests indicated repeated elevation of inflammatory markers, along with positive RF and ACPA levels (Table 1). Chest CT revealed an interval reduction in the lung abscess (Figure 2A). Arthrocentesis of knee demonstrated inflammatory synovial fluid with leukocytes without crystals. The patient was diagnosed with seropositive RA, and his symptoms improved with corticosteroid therapy. As the lung abscess continued to regress, biological therapy was initiated without infection recurrence (Figure 1).

**TABLE 1:** Laboratory test results at second admission.

Laboratory test	Value	Reference range
White blood cell count (×10^3^/μL)	136	33–86
Neutrophil count (×10^3^/μL)	111	13.2–49.6
Aspartate aminotransferase (U/L)	28	13–30
Alanine aminotransferase (U/L)	28	10–30
Lactate dehydrogenase (U/L)	149	124–222
Alkaline phosphatase (IU/L)	95	38–113
Total bilirubin (mg/dL)	0.88	0.40–1.50
Urea nitrogen (mg/dL)	20.4	8.0–20.0
Creatinine (mg/dL)	0.69	0.65–1.07
C‐reactive protein (mg/dL)	12.94	0.00–0.20
Immunoglobulin G (mg/dL)	2139	861–1747
Immunoglobulin A (mg/dL)	319	93–393
Immunoglobulin M (mg/dL)	98	33–183
Rheumatoid factor (IU/mL)	56	0–19
Anti‐citrullinated protein antibody (U/mL)	161	< 4.5
Matrix metalloproteinase‐3 (ng/mL)	399.1	36.9–121.0
Ferritin (ng/mL)	503	21–27.4

The patient had a history of treated salivary gland carcinoma and underwent annual positron emission tomography (PET) surveillance at a different hospital. Recent PET scan obtained 3 days before initial admission and reviewed retrospectively depicted new fluorodeoxyglucose uptake in the right shoulder and left hip joints, findings absent 1 year earlier and suggestive of preclinical inflammatory activity (Figure 2B).

## 3 Discussion

This case illustrates that RA may be clinically diagnosed during the acute phase of a respiratory infection, and that testing for RF and ACPA can facilitate early diagnosis. Although the association between RA and respiratory infections has been established as clinically important, its precise relationship remains unclear. This case provides clinically relevant insights into the relationship between infection‐related immune responses and the overt phase of seropositive RA.

According to the mucosal origin hypothesis, IgA‐type ACPAs locally produced at mucosal sites may precede and contribute to the emergence of IgG‐type ACPA that drive clinical arthritis. For example, smoking is a well‐established risk factor for RA and is strongly interrelated with IgA‐type ACPA production. Respiratory diseases have also been linked to an increased risk of RA, and chronic airway inflammation, such as bronchiectasis, is associated with increased RF and ACPA seropositivity [3]. However, these associations are generally correlated with chronic disease and prolonged preclinical stages. Contrarily, this case suggests that acute respiratory infections may accelerate the transition from latent autoimmunity to clinically overt RA. Notably, a Korean epidemiological study reported an increased incidence of new‐onset RA approximately 6–7 weeks after viral infection outbreaks [4], supporting the plausibility of the time course observed in our patient. Infectious stimuli may further promote arthritis by amplifying the inflammatory cytokine responses. Together, these observations suggest that an acute respiratory infection can act as an immunological trigger that amplifies preexisting mucosal autoimmunity, thereby precipitating the clinical onset of RA.

In older patients presenting with polyarticular pain following a respiratory infection, RA should be considered and RF and ACPA testing should be performed. The differential diagnosis is broad and includes septic arthritis, reactive arthritis, osteoarthritis and calcium pyrophosphate deposition. In older adults, joint symptoms may be dismissed as non‐specific or degenerative, potentially delaying the diagnosis of RA. Importantly, seropositive and seronegative RA differ in clinical course and underlying pathophysiology. In seropositive RA, particularly in patients with double seropositivity for RF and ACPA, a prolonged asymptomatic preclinical phase without overt joint damage may precede the development of clinical disease [5]. Moreover, the reported production of RF and ACPA within the inducible bronchus‐associated lymphoid tissue in the lungs supports a mechanistic link between mucosal immune responses and RA seropositivity. Together, when patients develop joint pain following a respiratory infection, the first critical step is to consider the possibility of RA. In such cases, it is reasonable to prioritize the likelihood of seropositive RA, and measurement of RF and ACPA represents a particularly appropriate diagnostic strategy.

Conclusively, this case demonstrates that RA can be diagnosed during the acute course of a respiratory infection and highlights the diagnostic value of RF and ACPA testing when new arthritis develops.

## Author Contributions

Y.A. and H.T. conceived of the case report and drafted the original manuscript. Y.A., H.T., K.H. and A.N. contributed to data interpretation and clinical assessment. T.A. and T.I. provided expert advice on clinical management and reviewed the manuscript. All the authors have read and approved the final version of this manuscript.

## Consent

The authors declare that written informed consent was obtained for the publication of this manuscript and accompanying images using the consent form provided by the Journal.

## Conflicts of Interest

The authors declare no conflicts of interest.

## Data Availability

Data sharing not applicable to this article as no datasets were generated or analysed during the current study.
